# OnlineStats.jl: A Julia package for statistics on data streams

**DOI:** 10.21105/joss.01816

**Published:** 2020-02-10

**Authors:** Josh Day, Hua Zhou

**Affiliations:** 1 Loon Analytics, LLC; 2 UCLA Biostatistics

## Abstract

The growing prevalence of big and streaming data requires a new generation of tools. Data often has infinite size in the sense that new observations are continually arriving daily, hourly, etc. In recent years, several new technologies such as Kafka (Apache Software Foundation, n.d.-a) and Spark Streaming (Apache Software Foundation, n.d.-b) have been introduced for processing streaming data. Statistical tools for data streams, however, are under-developed and offer only basic functionality. The majority of statistical software can only operate on finite batches and require re-loading possibly large datasets for seemingly simple tasks such as incorporating a few more observations into an analysis.

OnlineStats is a Julia (Bezanson, Edelman, Karpinski, & Shah, 2017) package for high-performance online algorithms. The OnlineStats framework is easily extensible, includes a large catalog of algorithms, provides primitives for parallel computing, and offers a weighting mechanism that allows new observations have a higher relative influence over the value of the statistic/model/visualization.

## Interface

Each algorithm is associated with its own type (e.g. Mean, Variance, etc.). The OnlineStats interface is built on several key functions from the OnlineStatsBase package. A new type must provide implementations of these functions in order to use the rest of the OnlineStats framework.

### Updating


OnlineStatsBase._fit!(stat, y)


The _fit! method determines how the statistic stat is updated with a single observation y. Each OnlineStat is a concrete subtype of OnlineStat{T}, where T is the type of a single observation. The fit!(stat::OnlineStat{T}, y::T) method simply calls _fit!. When fit!(stat::OnlineStat{T}, y::S) is called (where S is not a subtype of T), y is iterated through and fit! is called on each element.

#### Update Weights

Many OnlineStats incorporate a weight function that determines the influence of the next observation. For example, the online update for a mean *μ*^(*t*)^ given its current state *μ*^(*t*−1)^ and new observation *y*_*t*_ is

$$\mu^{\left(t\right)}=\left(1-t^{-1}\right)\mu^{\left(t-1\right)}+t^{-1}y_{t}.$$

OnlineStats generalizes this update to use weights that are a function of *t*:

$$\mu^{\left(t\right)}=[1-w(t)]\mu^{\left(t-1\right)}+w(t)y_{t}.$$

Therefore, for example, *w*(*t*) = *t*−1 returns the analytical mean and *w*(*t*) = λ (0 < λ < 1) returns an exponentially weighted mean.

### Merging


OnlineStatsBase._merge!(stat1, stat2)


The _merge! function merges the state of stat2 into stat1 and facilitates parallel computation. This function is optional to implement, as merging is not guaranteed to be well-defined for a given statistic/algorithm. The default definition prints out a warning that no merging occurred.

### Returning the State


OnlineStatsBase.value(stat, args...; kw...)


The value function returns the value of the estimator (optionally determined by positional arguments args and keyword arguments kw). Depending on the type, this may need to be calculated from its state. By default, this returns the first field of the type.


OnlineStatsBase.nobs(stat)


The nobs function returns the number of observations that the statistic has seen. By default this returns the n field from the algorithm’s type (stat.n).

## Example

The Mean type provides an easy-to-understand full example of how to implement a new algorithm. The update formula, as previously stated, is:

$$\mu^{\left(t\right)}=[1-w(t)]\mu^{\left(t-1\right)}+w(t)y_{t}.$$

The merge formula for two means, $\mu_{1}^{\left(t\right)}$ and $\mu_{2}^{\left(s\right)}$, generalizes the above equation to:

$$\mu_{merged}^{\left(t+s\right)}=[1-s/(t+s)]\mu_{1}^{\left(t\right)}+[s/(t+s)]\mu_{2}^{\left(s\right)}.$$

Converting these formulas into Julia code, we get the following implementation. Note that Mean is parameterized by the data type that stores the mean value so that non-standard types such as complex numbers can be used.


mutable struct Mean{T,W} <: OnlineStat{Number}
  : :T
  weight: :W
  n: :Int
end
function Mean(T: :Type{<:Number} = Float64; weight = inv)
  Mean(zero(T), weight, 0)
end
function _fit!(o: :Mean{T}, x) where {T}
  o.n += 1 
  w = T(o.weight(o.n)) 
  o. += w * (x - o.)
end
function _merge!(o: :Mean, o2: :Mean)
  o.n += o2.n
  o. += (o2.n / o.n) * (o2. - o.)
end

